# GCA-Trans: Global Context-Aware Transformer for Robust Transparent Object Segmentation in Robotic Environments

**DOI:** 10.3390/jimaging12050212

**Published:** 2026-05-16

**Authors:** Deping Li, Zujian Dong, Zilong Yang, Ka-Kui Li, Yushen Huang

**Affiliations:** 1 School of Intelligent Systems Science and Engineering, Jinan University, Zhuhai 510632, China; lideping@jnu.edu.cn (D.L.); 13265195268@163.com (K.-K.L.); 2 Department of Informatics, Karlsruhe Institute of Technology, 76131 Karlsruhe, Germany; zilong.yang@student.kit.edu; 3 Department of Mathematics, South China University of Technology, Guangzhou 510641, China; 202367400081@mail.scut.edu.cn

**Keywords:** transparent object segmentation, semantic segmentation, scene understanding, transformer, convolutional neural network

## Abstract

Transparent object segmentation plays a critical role in indoor and outdoor scene understanding, particularly driven by the rapid advancements in autonomous driving and robotics. However, this task presents significant challenges due to the lack of distinct texture and chromatic features in transparent objects, causing their appearance to blend into the background. Existing methods face inherent architectural limitations: CNNs are restricted by limited receptive fields, while Transformer-based methods may inadvertently suppress the weak feature details of transparent surfaces due to the inherent low-pass filtering property of self-attention mechanisms, treating them as background noise. Consequently, these approaches struggle to consistently segment transparent objects across diverse scales, failing to preserve both fine details and large-scale structures. To address these limitations, we propose the Global Context-Aware Transformer (GCA-Trans). Specifically, we design a Multi-scale Context Mining (MCM) module that leverages parallel dilated convolutions with varying receptive fields to simultaneously extract features at multiple scales. This design allows the model to capture and fuse fine-grained local details (e.g., edges and textures) with coarse-grained global spatial context (e.g., overall object shapes), ensuring robust segmentation performance for transparent objects of varying scales. Extensive experiments on four benchmark datasets demonstrate that GCA-Trans sets a new state of the art, achieving significant improvements of 2.53% mIoU on Trans10K-v2, 2.1% IoU on RGB-D GSD, 2.2% IoU on GDD, and 1.9% IoU on GSD, validating the effectiveness and robustness of our approach.

## 1. Introduction

With the rapid advancement of autonomous driving and robotic systems, precise scene understanding has become crucial. This capability is particularly vital in autonomous chemistry and biomedical laboratories [1], where robots are tasked with grasping and manipulating various experimental instruments. However, the prevalence of transparent materials in these settings, ranging from reagent containers to glass doors, presents specific perception challenges. Unlike opaque objects, transparent surfaces often lack distinct texture and chromatic features, making them difficult to distinguish from the background. Furthermore, their unique optical properties violate the Lambertian assumption [2] fundamental to standard 3D sensors. Since these surfaces predominantly transmit light or exhibit specular reflections, projected infrared patterns may penetrate the object or deflect away, potentially leading to incomplete depth maps [2,3]. These limitations pose significant challenges to autonomous robots, forcing them to rely on subtle visual cues to accurately manipulate glassware and detect transparent boundaries, such as distinguishing between a closed glass cabinet door and an open space during reagent retrieval.

In order to tackle the challenges posed by transparent object segmentation, researchers have explored various methodologies. Due to the high cost and deployment constraints of depth sensors, the community has largely shifted towards RGB-based solutions [2]. Existing methods can be broadly categorized into CNN-based and Transformer-based approaches. CNN-based works have primarily focused on exploiting specific visual cues to compensate for the lack of texture. For instance, TransLab [4] and EBLNet [5] utilized boundary cues to distinguish transparent regions from the background, while GDNet [6] and GSDNet [7] introduced large-field contextual integration and reflection priors to reduce ambiguity. Recently, Lin et al. [3] proposed a novel glass surface detection framework combining RGB and depth information, referred to as RGB-D GlassNet. However, these early attempts leveraging boundaries or reflections often fail to correctly distinguish the semantic categories of transparent objects.

Distinct from CNN architectures that primarily focus on local cues, Transformer-based methods utilize self-attention mechanisms to capture long-range dependencies. As a pioneer, Trans2Seg [8] proposed a Transformer encoder–decoder architecture, providing a global receptive field for dynamic mask prediction. Subsequently, Trans4Trans [9] employed a symmetrical Transformer framework to harvest multi-scale feature representations from dense partition embeddings. More recently, TOSQ [10] and To-Former [11] have surpassed previous state-of-the-art methods on the Trans10K-v2 dataset; specifically, TOSQ utilizes a query-based dictionary lookup mechanism to enhance feature representation, while To-Former incorporates edge-enhanced transformers. Although recent Transformer-based methods attempt to address the suppression of fine-grained local details by focusing on boundaries, they still lack sufficient attention to internal fine-grained local details [12], such as surface textures, leading to suboptimal performance on large-scale transparent objects.

To remedy the suboptimal performance of Transformer-based methods on large-scale transparent objects, which is primarily attributed to the low-pass filtering nature of multi-head self-attentions (MSAs) that tends to suppress high-frequency surface signals, such as faint reflections and subtle textures [12], we propose a Multi-scale Context Mining (MCM) strategy to effectively capture fine-grained local details. By employing parallel dilated convolutions with varying rates, MCM effectively expands the receptive field to capture multi-scale context. To mitigate the subsequent ’gridding effect’ introduced by dilation, we further integrate multi-scale pooling. Crucially, this design preserves fine-grained local details, such as boundaries, faint reflections, and subtle textures, while capturing coarse-grained global spatial context. Building upon this design, the Global Context-Aware Transformer (GCA-Trans) is proposed. The framework utilizes PVT-v2 [13] as the backbone to extract multi-scale features. The MCM module is deployed at each decoder stage to independently refine these features, after which the enhanced features are upsampled and fused, passing through a small convolution head to generate the final precise segmentation map.

The main contributions of this paper are summarized as follows:

(1) To overcome the suboptimal performance of existing methods in handling the significant scale variation in transparent objects, we propose the Global Context-Aware Transformer (GCA-Trans) framework. This framework achieves consistent segmentation accuracy across diverse scales, ensuring reliability in complex scenarios.
(2) To address the inherent deficiency of Transformers in capturing fine-grained details, we design a Multi-scale Context Mining (MCM) module within the decoder. It simultaneously captures fine-grained local details, such as boundaries, subtle textures, and faint reflections, as well as coarse-grained global spatial context.
(3) Extensive experiments demonstrate that GCA-Trans achieves state-of-the-art performance on four challenging transparent object segmentation datasets, validating the effectiveness of the MCM module and the capability of the GCA-Trans framework in segmenting transparent objects of varying scales. Furthermore, additional tests on general scenes validate its robust performance and strong generalization ability.

## 2. Related Works

### 2.1. Sensors for Robotic Perception of Transparent Objects

Robotic perception of transparent objects relies on diverse sensor modalities to mitigate challenges arising from weak surface features and the violation of the Lambertian assumption. Active sensing devices, such as RGB-D cameras (e.g., Intel RealSense), utilize IR laser projectors to generate textures for depth densification [14,15,16,17]. While effective, they frequently suffer from background depth misestimation and missing depth values due to refraction. To address this challenge, Fan et al. [18] introduced TDCNet, a novel dual-branch hybrid network combining CNNs and Transformers, specifically tailored for transparent object depth completion. Stereo vision systems, such as KeyPose [19], employ cameras like the ZED to simulate binocular parallax for pose estimation, yet they often struggle in texture-less or low-light conditions. Light-field cameras capture both light intensity and directionality to enhance shape analysis for recognition and segmentation [20,21,22]; however, their application is often hindered by high computational intensity. Polarization cameras, such as the Blackfly S, excel at eliminating glare and extracting material properties, often outperforming standard RGB sensors in segmentation tasks [23,24], but come with significantly higher hardware costs. RGB-T solutions integrate thermal imaging to adapt to low-light environments and reduce texture interference [25], though they are constrained by varying costs and typically lower resolutions. Furthermore, tactile sensors like GelSight provide precise contact-based physical data to complement visual perception [26], but their utility is limited to small, local detection areas. While these specialized sensors offer distinct advantages, their limitations in cost, deployment complexity, and environmental adaptability have driven the search for more accessible, general-purpose solutions.

### 2.2. CNN-Based Methods

Following the widespread adoption of Convolutional Neural Networks (CNNs), numerous approaches have been developed to exploit specific visual cues of transparent objects from standard RGB sensor data. Early works adapted general object detection frameworks to this domain; for instance, Madessa et al. [27] employed Mask R-CNN to detect individual transparent instances. To address the unique optical distortions captured by visual sensors, Chen et al. [28] proposed TOM-Net, a framework learning the refractive flow for transparent object matting. Similarly, addressing the reflection property, Mei et al. [6] designed GDNet to integrate global context with reflection priors for glass detection. Recognizing that transparent objects often lack texture but possess strong edge features, Xie et al. [4] introduced TransLab, utilizing boundary cues to improve segmentation accuracy. Building on this, He et al. [5] proposed EBLNet with a differential module to output finer boundary cues, while Lin et al. [7] extracted multi-scale boundary features to differentiate glass regions from reflections. In terms of real-time processing for industrial or robotic sensors, Xu et al. [29] optimized DeepLabV3+ to mitigate segmentation inaccuracies caused by the similarity between transparent objects and their background. More recently, researchers have sought to fuse data from different sensor modalities. Lin et al. [3] observed that the transmission of active light through glass often results in blank regions in depth maps produced by 3D sensors. Leveraging this sensor-specific characteristic, they proposed a framework combining RGB features with these depth invalidity cues to robustly detect glass surfaces.

### 2.3. Transformer-Based Methods

Propelled by the success of Vision Transformers (ViTs) [30], current research increasingly employs self-attention mechanisms to capture the long-range dependencies that are challenging for conventional CNNs. To address the locality constraints of CNNs, Xie et al. [8] introduced Trans2Seg, a pioneering framework that leverages self-attention to extract global environmental features for fine-grained transparent object segmentation. Building on this global modeling capability, subsequent works have focused on optimizing feature interaction. Zhang et al. [9] proposed a lightweight Transformer Parsing Module (TPM) to perform multi-scale feature interpretation, enabling the segmentation of both general and transparent objects. Similarly, Hu et al. [31] developed a ViT-based architecture called TGSNet, which associates multilevel receptive fields to retain comprehensive feature information across different scales. Recognizing the importance of boundary details, Chen et al. [32] proposed EG-Trans to enhance edge information within the transformer architecture while integrating global context. In a related approach, Chen et al. [11] introduced To-Former, featuring an edge-enhanced multi-head self-attention mechanism that incorporates multi-scale separable convolution and pooling. More recently, Ma et al. [10] proposed TOSQ by designing a Query Parsing Module (QPM) that innovatively formulates segmentation as a dictionary lookup problem, further advancing the efficiency of transformer-based decoding.

## 3. Methodology

**Figure 1:**
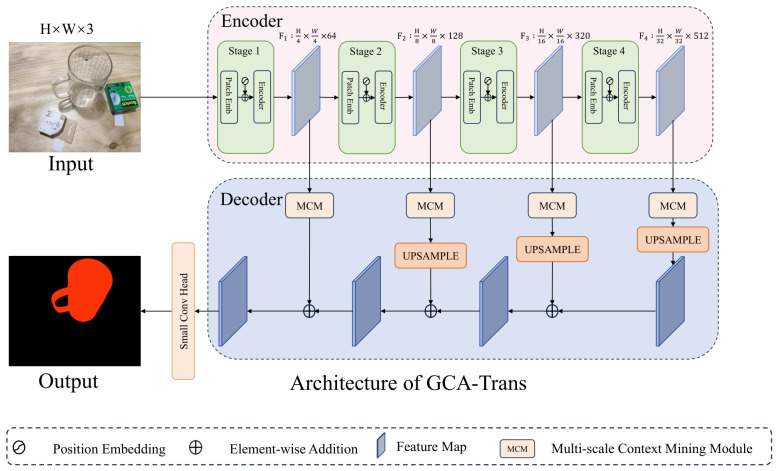
The overall architecture of the proposed GCA-Trans framework. It employs PVT-v2 as the backbone encoder to extract hierarchical features, followed by a decoder integrating our Multi-scale Context Mining (MCM) modules at each stage to refine features before the final prediction.

Most existing methods disproportionately emphasize boundary information while neglecting the subtle texture details on transparent object surfaces. This oversight limits the extraction of discriminative features, consequently leading to inferior accuracy in both semantic classification and segmentation. As illustrated in Figure 1, we propose GCA-Trans to address this issue.

### 3.1. Overview

The overall architecture of GCA-Trans is depicted in Figure 1. PVT-v2 [13] is adopted as the backbone to extract hierarchical multi-scale features across four stages. These feature maps are subsequently fed into the proposed Multi-scale Context Mining (MCM) module for contextual refinement. Following this enhancement, the output features from each stage are upsampled to a unified resolution (matching the first stage). Finally, the multi-level features are fused via element-wise addition and processed by a convolutional layer to generate the final segmentation map.

### 3.2. Multi-Scale Context Mining (MCM) Module

**Figure 2:**
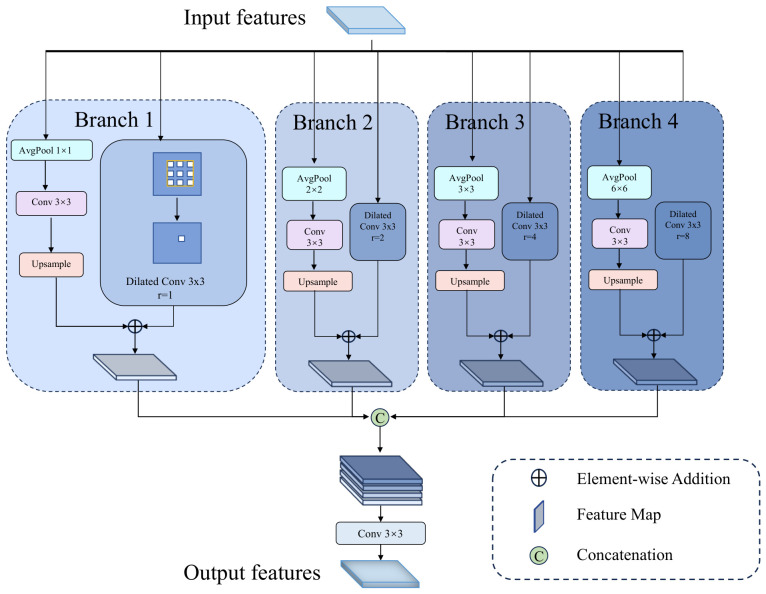
The detailed structure of the Multi-scale Context Mining (MCM) module. It consists of four parallel branches, each explicitly coupling a dilated convolution path (with varying dilation rates *r*) and a multi-scale pooling path (with varying pooling sizes) via element-wise addition to capture both local details and global context.

As illustrated in Figure 2, the MCM module handles scale variations via four parallel branches. Each branch comprises two complementary streams: a dilated convolution stream that leverages varying dilation rates to extract multi-scale features via distinct receptive fields, and a pooling stream that employs diverse pooling scales to preserve coarse-grained global spatial context, effectively mitigating the ‘gridding effect’ of dilated convolutions.

**Dilated Convolution Path.** To capture multi-scale representations, dilated convolutions with varying dilation rates $r_{i}\in \left\{1,2,4,8\right\}$ [33] are employed. This design is motivated by the unique visual attributes of transparent objects, particularly their subtle reflection textures and distinctive edge information. Specifically, branches with smaller dilation rates (e.g., r=1,2) focus on extracting fine-grained local details within limited receptive fields, preserving intricate cues like surface textures, reflections, and object boundaries. In contrast, branches with larger dilation rates (e.g., r=4,8) exponentially expand the receptive field to aggregate wider-range context, allowing the model to infer overall object shapes based on environmental priors. For an input feature map $F\in R^{C\times H\times W}$, the output of this path is denoted as ${\mathrm{Conv}}_{r_{i}}\left(F\right)$.

**Multi-scale Pooling Path.** To mitigate the ‘gridding effect’ [34] of dilated convolutions and enhance the representation of coarse-grained global spatial context, we introduce a parallel pooling path inspired by PSPNet [35]. Adaptive average pooling with output sizes S={1×1,2×2,3×3,6×6} is employed to aggregate dense context from varying sub-regions, effectively compensating for local information loss. The pooled features are then projected via a 3×3 convolution and upsampled to the input feature resolution.

**Feature Fusion.** While traditional modules like ASPP [36] and PPM [35] treat dilated convolutions and multi-scale pooling as completely independent parallel branches that are only aggregated at the very end, our MCM explicitly couples them early on. By pairing a specific receptive field (via dilation rate) with a corresponding spatial density (via pooling size) within each individual branch prior to the final cross-scale concatenation, MCM ensures that fine-grained local details (such as textures and object boundaries) and coarse-grained global spatial context are dynamically bound at each specific scale. To implement this, local details from the dilated path and dense context from the pooling path are effectively fused within each branch via element-wise addition. Formally, the output $F_{i}\in R^{\frac{C}{4}\times H\times W}$ of the *i*-th branch is computed as:

(1) $$F_{i}={\mathrm{Conv}}_{r_{i}}\left(F\right)+H_{\mathrm{up}}\left({\mathrm{Conv}}_{3\times 3}\left({\mathrm{AvgPool}}_{s_{i}}\left(F\right)\right)\right),$$

where ${\mathrm{Conv}}_{r_{i}}\left(\cdot \right)$ denotes a dilated convolution with rate $r_{i}$ projecting the input to C/4 channels, ${\mathrm{AvgPool}}_{s_{i}}\left(\cdot \right)$ performs adaptive average pooling with output size $s_{i}$, ${\mathrm{Conv}}_{3\times 3}\left(\cdot \right)$ applies a 3×3 convolution with a padding of 1, and $H_{\mathrm{up}}\left(\cdot \right)$ represents bilinear upsampling. Finally, to synthesize the multi-scale cues extracted from all branches, the outputs $\left\{F_{1},F_{2},F_{3},F_{4}\right\}$ are concatenated and fused through a 3×3 convolution layer, thereby integrating the fine-grained local details and coarse-grained global spatial context into a unified holistic feature map:

(2) $$F_{\mathrm{out}}={\mathrm{Conv}}_{3\times 3}\left(\mathrm{Concat}(F_{1},F_{2},F_{3},F_{4})\right),$$

where ${\mathrm{Conv}}_{3\times 3}$ maps the concatenated features to $C_{out}$ channels, yielding a representation that is both locally precise and globally coherent. This specific kernel size is empirically chosen as it provides an optimal trade-off: it is large enough to effectively capture local spatial correlations and smooth any potential artifacts from the multi-path aggregation yet compact enough to maintain computational efficiency.

## 4. Experiments and Results

### 4.1. Datasets and Evaluation Metrics

#### 4.1.1. Datasets

**Trans 10K-v2 [8]:** This dataset is officially partitioned into 5000, 1000, and 4428 images for training, validation, and testing, respectively. The images feature a standardized resolution of 835×1113. It provides fine-grained annotations across 11 semantic categories, including shelf, jar/tank, freezer, window, glass door, eyeglass, cup, wall, glass bowl, water bottle, and storage box.
**GDD [6]:** Focused on glass detection in real-world environments, this dataset comprises 3916 image-mask pairs. It covers diverse daily-life scenarios, including 2827 images from indoor scenes (e.g., bathrooms, offices) and 1089 images from outdoor scenes (e.g., streets, malls). Following the official split protocol, we utilize 2980 images for training, while the remaining 936 images are reserved for testing.
**GSD [7]:** This dataset consists of 4012 real-world images paired with precise pixel-level masks for glass surfaces. It is characterized by high diversity in object scale, covering closeup, medium, and long shots across various scenes. For the experimental setup, the dataset is partitioned into a training set of 3202 images and a test set of 810 images.
**RGB-D GSD [3]:** This dataset comprises a total of 3009 image pairs, featuring both RGB images and their corresponding depth maps. Following the standard evaluation protocol, the dataset is partitioned into a training set of 2400 images and a testing set of 609 images.

**Figure 3:**
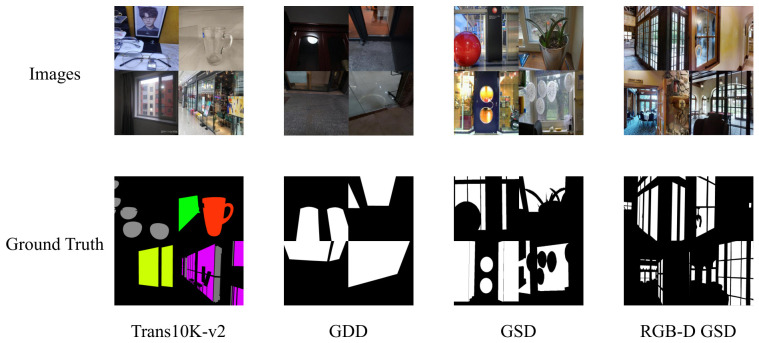
Representative example images and their corresponding ground truth masks from the four evaluated benchmark datasets.

To provide a more intuitive understanding of the task complexity and the characteristics of the data, we summarize the statistical details of all four evaluated datasets in Table 1. Furthermore, representative sample images and their corresponding ground truth masks are visualized in Figure 3. As illustrated, these datasets encompass a wide variety of transparent objects, ranging from small indoor glass cups to large outdoor glass doors captured under diverse lighting conditions.

#### 4.1.2. Evaluation Metrics

GCA-Trans is evaluated on four datasets categorized into two tasks. For multi-class semantic segmentation on **Trans10K-v2** [8], we assess performance using **mIoU**, Pixel Accuracy (**Acc**), and model complexity (**GFLOPs**). For binary glass detection on **RGB-D GSD** [3], **GDD** [6], and **GSD** [7], we employ four standard metrics: Intersection-over-Union (**IoU**), F-measure (**$F_{\beta }$**), Mean Absolute Error (**MAE**), and Balance Error Rate (**BER**). All datasets follow the standard training, validation, and testing splits provided in their original papers.

### 4.2. Implementation Details

Implemented in PyTorch 1.8.0 with CUDA 11.2,GCA-Trans is trained on two NVIDIA 2080Ti GPUs (Nvidia Corporation, Santa Clara, CA, USA) (batch size of 4 per GPU) for 100 epochs. AdamW [37] is utilized as the optimizer ($lr=1e^{-4}$, weight decay = $1e^{-4}$, $\epsilon =1e^{-8}$) combined with a poly scheduling strategy (power 0.9) [38]. For the objective function, we employ the standard Cross-Entropy Loss without class weighting. The PVT-v2 backbone is initialized using ImageNet-1K pretrained weights. In the decoder (MCM module), Batch Normalization and ReLU activation are applied after each convolutional layer to ensure stable training. Images are resized to 512×512 for all experiments. During training, no complex data augmentation is applied; instead, augmentation is restricted to basic resizing and normalization. For evaluation, the best-performing model is selected based on the highest mIoU achieved on the validation set. During inference, we adopt a single-scale testing strategy without test-time augmentation. For binary detection tasks, a standard threshold of 0.5 is applied to generate the final masks. To ensure reproducibility, all experiments are conducted with a fixed random seed 1024. Computational complexity (GFLOPs) is evaluated at 512×512.

### 4.3. Compared with State-of-the-Art Methods

**Baseline Evaluation Protocols.** For the multi-class semantic segmentation results in Table 2, RGB-D GlassNet and GDNet were re-trained from scratch under identical experimental settings to our proposed GCA-Trans (i.e., same data splits, resolution, and augmentations). ToFormer(B) was re-implemented strictly following the configurations detailed in its original paper. The performance metrics for the remaining baseline models are cited directly from the comprehensive benchmarks established in the TOSQ [10] and Trans4Trans [9] publications. For the binary glass detection tasks in Table 3, both Trans4Trans-M and our PVT-v2 baseline were re-trained under our unified settings to ensure strict comparability.

As shown in Table 2, GCA-Trans-b4 achieves top performance on Trans10K-v2 with **80.00%** mIoU, outperforming TOSQ-256 [10] by **2.5%**. Notably, GCA-Trans demonstrates consistent superiority across diverse categories containing transparent objects of varying sizes, thereby validating the effectiveness of our approach in multi-scale segmentation. We focus on b3 and b4 variants as larger models yield marginal gains.

As detailed in Table 2, we comprehensively compare the computational cost (GFLOPs) of GCA-Trans against analogous methods. We acknowledge that lightweight models like Trans4Trans-M and TOSQ-256 possess a distinct advantage in computational overhead. In contrast, our GCA-Trans-b4 prioritizes segmentation precision, trading a higher computational complexity for a noticeable 2.53% mIoU improvement.

**Figure 4:**
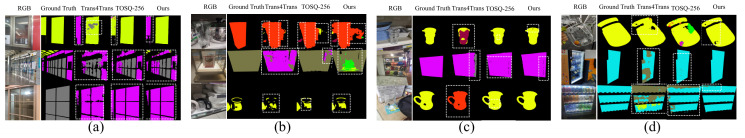
Qualitative analysis on Trans10K-v2 test set. The white dotted boxes highlight specific local regions of interest for visual comparison. (**a**,**b**) show failure cases where all evaluated models produce incomplete or inaccurate segmentation masks. In (**c**,**d**), our GCA-Trans produces more precise segmentation masks compared to Trans4Trans and TOSQ-256.

To provide a more intuitive assessment, we present qualitative comparisons in Figure 4. As illustrated in Figure 4c,d, our GCA-Trans produces more precise segmentation masks with fewer false negatives compared to both the representative Trans4Trans and the stronger baseline TOSQ-256. However, Figure 4a,b also highlight persistent challenges; although the models successfully localize the transparent regions, they occasionally misclassify the specific semantic categories of these objects, or erroneously segment adjacent specular reflections as transparent materials.

**Figure 5:**
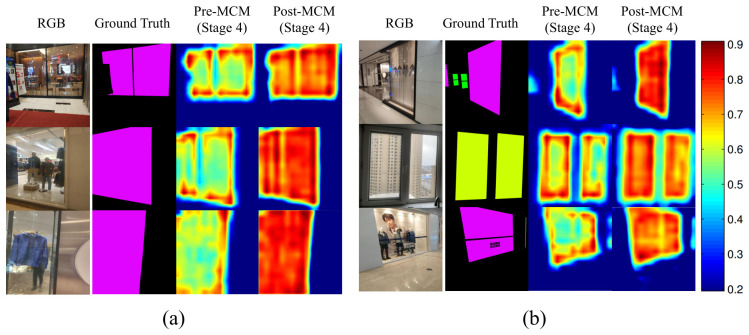
Visual comparison of feature heatmaps generated via Grad-CAM. (**a**,**b**) compare the features from the backbone’s 4th stage (Pre-MCM) with the refined features output by the MCM module (Post-MCM). The color bar on the right indicates the activation magnitude, where higher values denote regions of higher model attention.

Furthermore, to visualize the internal feature refinement process, we provide feature heatmaps generated via Grad-CAM in Figure 5. As illustrated, the heatmaps show a shift from attention concentrated on boundaries (Pre-MCM) to a comprehensive representation covering the object’s interior (Post-MCM). This demonstrates that our method successfully captures subtle texture details, balancing attention between distinctive edges and the complete transparent surface.

For the binary glass detection tasks, we employ the GCA-Trans-b4 variant for comparison. As shown in Table 3, GCA-Trans-b4 demonstrates great competitiveness, achieving the best performance across the majority of metrics on all three datasets (RGB-D GSD, GDD, and GSD). Although the RGB-D GSD dataset provides depth information, our GCA-Trans relies exclusively on RGB imagery as input for all experiments. To ensure a fair comparison with multimodal methods like RGB-D GlassNet, we evaluate our RGB-only model against their reported results. Notably, GCA-Trans significantly outperforms the PVT-v2 baseline, confirming the effectiveness of our proposed MCM module. These consistent improvements across diverse datasets demonstrate the strong generalization capability of our model in complex scenarios.

**Figure 6:**
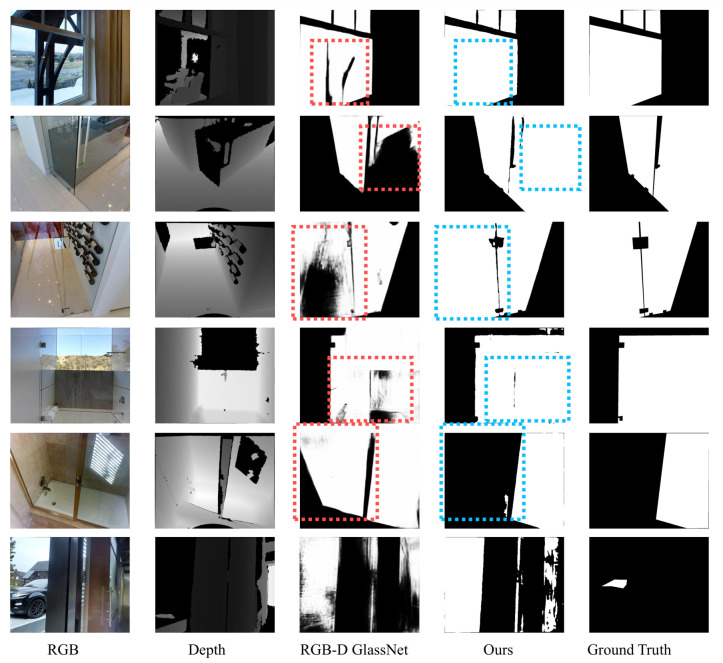
Visual comparison of our method with state-of-the-art methods on images from RGB-D GSD dataset. Red dashed boxes highlight regions where the SOTA model produces inaccurate or fragmented predictions, while blue dashed boxes indicate the corresponding areas where our method achieves significant improvements and produces more precise segmentation masks. Additionally, the bottom row shows a failure case for both models misidentifying a hollow region as glass.

To provide a more intuitive comparison, we visualize the qualitative segmentation results on the RGB-D GSD dataset in Figure 6. Specifically, we compare our GCA-Trans against the state-of-the-art method RGB-D GlassNet [3]. Our GCA-Trans produces more precise segmentation masks with significantly fewer holes and fragmented regions compared to RGB-D GlassNet.

A challenging case is shown in the second to last row, which contains a half-opened glass door. While RGB-D GlassNet incorrectly predicts the open area as glass, our method correctly separates the glass from the background. Furthermore, the bottom row illustrates a failure case for both methods, where the models incorrectly identify a hollow region as glass.

### 4.4. Generalization and Robustness Analysis

#### 4.4.1. Cross-Dataset Evaluation on VGSD

In real-world robotic deployment, perception systems must maintain robustness within dynamic and unpredictable environments, rather than overfitting to static training distributions. To rigorously evaluate the generalization capability and robustness of our proposed method under such conditions, we perform zero-shot segmentation using the trained model on the test set of VGSD-D [46].

Specifically, we utilize individual training sets (GDD, GSD, and RGB-D GSD) to evaluate our model (GCA-Trans-b4) under a strict single-source zero-shot setting. Notably, the target VGSD-D dataset is large-scale, containing 12,315 training frames (from 192 videos) and 6851 testing frames. In contrast, our source training sets (GDD, GSD, and RGB-D GSD) are significantly smaller, each containing fewer than 3000 images. Subsequently, we directly evaluated the model on the test set of the VGSD-D dataset [46] without any fine-tuning, using only the RGB frames while ignoring the depth maps. To ensure experimental rigor, we confirm that there is no data overlap between these source datasets and the target VGSD-D dataset. Unlike the training data, VGSD-D consists of sequential frames extracted from videos, introducing motion blur and dynamic lighting changes that mimic actual robotic operation scenarios.

Regarding the cross-dataset evaluation in Table 4, the baseline results are cited directly from the original VGSD-D [46] paper.

As shown in Table 4, our method achieves superior performance across all key metrics. These results demonstrate promising generalization capability of GCA-Trans, enabling effective transparent object perception in dynamic environments even without exposure to domain-specific training data.

#### 4.4.2. Evaluation on General Semantic Segmentation

To evaluate the versatility of our proposed decoder beyond transparent objects, we trained and tested GCA-Trans-b4 on the Cityscapes dataset, following the experimental settings of TOSQ [10].

As shown in Table 5, our method demonstrates superior performance in general scenes. This validates the effectiveness and transferability of our decoder architecture, demonstrating its capability to handle complex environmental conditions in general semantic segmentation tasks.

### 4.5. Ablation Studies

#### 4.5.1. Effectiveness of GCA-Trans

To validate the effectiveness and generalizability of our proposed framework, we establish strong baselines using both the PVT-v1 [57] and PVT-v2 [13] series as backbones. For comparison, we equip these backbones with classic multi-scale context modules, including ASPP [36] and PPM [35]. All models are trained and evaluated under identical settings on the challenging Trans10K-v2 dataset.

**Figure 7:**
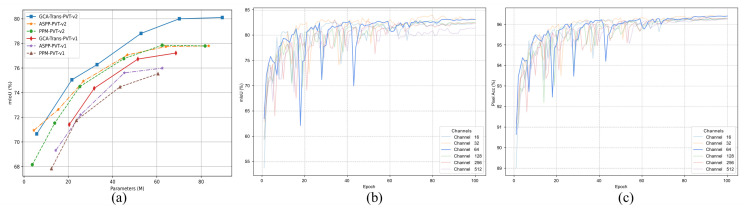
Performance and ablation analysis. (**a**) mIoU vs. Parameters comparison of our GCA-Trans against ASPP and PPM across PVT-v1 and PVT-v2 backbone series. (**b**) mIoU and (**c**) pixel accuracy curves of 6 MCM channel settings.

As illustrated in Figure 7a, we comprehensively compare the mIoU and model parameters of our GCA-Trans against models equipped with ASPP and PPM. The results demonstrate that our approach generally achieves a more favorable performance-to-parameter trade-off across both PVT-v1 and PVT-v2 backbone series. Compared to the ASPP and PPM variants, GCA-Trans attains improved mIoU scores across evaluated scale configurations with a reasonable increase in parameters, confirming the effectiveness of our architecture.

#### 4.5.2. Impact of MCM Components and Channels

The design of the core MCM module is verified in Table 6. Comparisons against single-path variants (“Only Dilated” or “Only Pooling”) confirm that both paths are essential for performance. The performance drop with ASPP [36] validates our design of explicitly decoupling texture and semantic cues. Furthermore, by varying the output channel dimension $C_{out}$ from 16 to 512, we find that $C_{out}=64$ yields the best performance with an mIoU of 78.81%. As presented in Table 6, increasing the channel dimension beyond this point does not lead to further improvements; instead, we observe a slight performance decline. This trend, as visualized in Figure 7b,c, suggests that excessive model capacity (e.g., $C_{out}\geq 128$) may lead to redundant parameters that do not further enhance feature representation, resulting in a performance saturation. Moreover, the computational complexity becomes exceedingly large with the increase in the channel number, significantly reducing efficiency. Therefore, we adopt $C_{out}=64$ as the optimal configuration to balance accuracy and efficiency.

### 4.6. Detailed Analysis

#### 4.6.1. Robustness to Object Scales

To comprehensively evaluate the robustness of GCA-Trans across diverse object scales, we conducted a quantitative analysis on the Trans10K-v2 test set. Specifically, we partitioned the transparent objects into five equal intervals from 0% to 100% based on their area proportion relative to the total image area.

**Figure 8:**
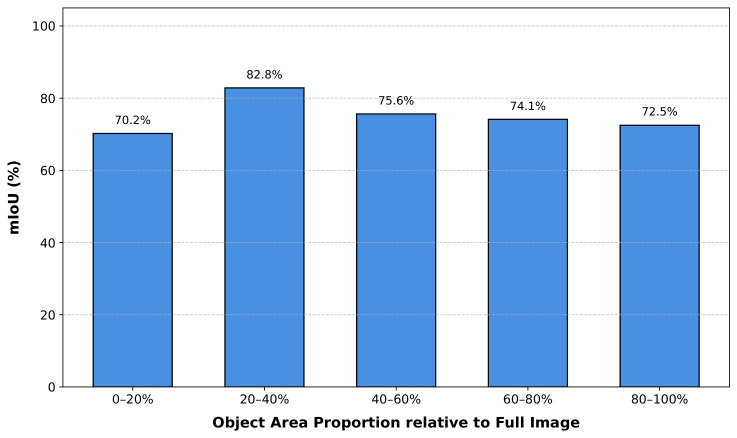
Segmentation Performance across Object Scales.

As illustrated in Figure 8, GCA-Trans-b4 achieves consistent and high-quality segmentation results across all scale ranges. Notably, the model maintains a high mIoU of 70.2% for small-scale objects (0–20%) and 72.5% for large-scale structures (80–100%), reaching a peak of 82.8% in the 20–40% interval. These results demonstrate that our proposed method achieves great segmentation performance for transparent objects across diverse scales.

#### 4.6.2. Analysis Under Weak Lighting and Semantic Complexity

To further explore the segmentation performance of GCA-Trans in challenging environments, we conduct an analysis under weak lighting conditions and multi-category scenarios.

**Figure 9:**
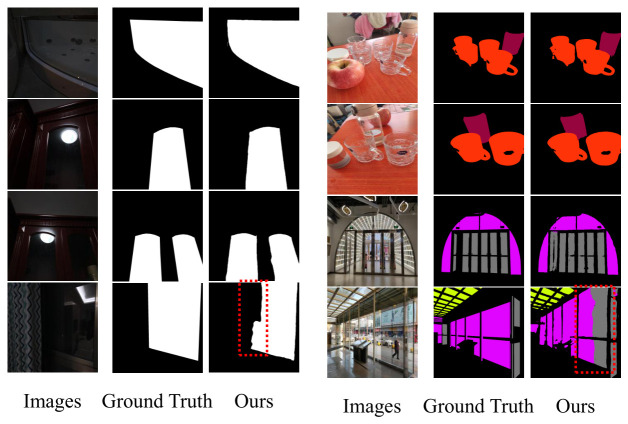
Performance analysis of GCA-Trans under weak lighting (**left** panel) and multi-category complex scenes (**right** panel). The bottom row presents failure cases, where the red dashed boxes highlight the specific regions of inaccurate predictions.

Figure 9 illustrates the visual results across these diverse settings. As shown in the first three rows, our model performs well in both low-contrast dark environments and complex scenes containing multiple transparent objects. However, the failure cases in the bottom row reveal that under extremely low illumination where distinct visual features are virtually absent, the model’s performance remains limited. Notably, even when GCA-Trans successfully detects the presence of a transparent object, performing accurate semantic classification remains a significant challenge due to the lack of discriminative texture and chromatic cues in such extreme conditions.

#### 4.6.3. Inference Efficiency for Robotic Deployment

To evaluate the practical feasibility of GCA-Trans for real-world robotic systems, we conduct a comprehensive inference efficiency analysis across various backbone scales. All measurements are performed on a single NVIDIA 2080Ti GPU with a standardized input resolution of 512×512 and a batch size of 1.

As summarized in Table 7, our proposed framework demonstrates competitive real-time performance. Specifically, the lightweight GCA-Trans-b0 achieves an exceptional inference speed of 68.12 FPS with a low latency of 14.68 ms. For the GCA-Trans-b4 variant, which offers the optimal balance between accuracy and complexity, it maintains a near-real-time processing speed of 21.74 FPS with a peak GPU memory consumption of 4496.33 MB and RAM usage of 3757.29 MB. These metrics demonstrate that GCA-Trans has great potential for integration into robotic perception pipelines, maintaining superior performance while ensuring low-latency response in dynamic environments.

#### 4.6.4. Failure Case Analysis

**Figure 10:**
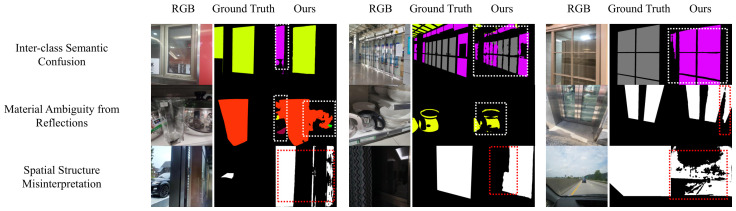
Typical failure cases of GCA-Trans. Dashed boxes highlight inaccurate predictions across three representative challenges (white dashed boxes denote multi-class semantic segmentation, while red dashed boxes denote binary glass detection): (**Top**) Inter-class Semantic Confusion among objects with similar backgrounds; (**Middle**) Material Ambiguity caused by strong specular reflections; and (**Bottom**) Spatial Structure Misinterpretation, including incomplete masks and false positives in hollow regions.

To provide a comprehensive understanding of the limitations of the proposed GCA-Trans framework, we categorize and analyze the typical failure cases observed during our experiments, as illustrated in Figure 10.

**Inter-class Semantic Confusion:** As shown in the top row, the model occasionally struggles to differentiate between distinct semantic categories of transparent objects, such as confusing a glass window with a glass door. This primarily occurs because both objects often share identical indoor backgrounds and exhibit similar visual transmission properties. Although our proposed MCM module effectively extracts multi-scale context, relying solely on 2D RGB appearance without additional 3D geometric constraints or spatial priors makes it challenging to accurately resolve such functional semantic ambiguities.

**Material Ambiguity from Reflections:** The middle row demonstrates instances where non-transparent objects with highly reflective or specular surfaces, such as polished metals, are erroneously segmented as transparent. The model may occasionally over-rely on these visual cues like sharp edges and bright specular highlights, mistakenly interpreting the intense specular reflections of metallic surfaces as the refractive properties typical of glass.

**Spatial Structure Misinterpretation:** The bottom row highlights errors related to complex spatial topologies, including incomplete mask generation and false-positive predictions in hollow regions. In such scenarios, the background visible through the empty space is visually indistinguishable from the background transmitted through the adjacent glass. The pure RGB framework struggles to constrain the segmentation mask exclusively to the physical transparent surface, leading to regional over-segmentation.

These specific failure cases underscore the inherent limitations of relying exclusively on RGB sensors in highly ambiguous environments, suggesting that integrating multi-modal sensing or physical geometry priors could be a promising direction for future research.

## 5. Limitations and Future Work

Although GCA-Trans demonstrates superior accuracy and strong generalization in diverse environments, several limitations provide avenues for future research.

### 5.1. Ambiguous Scenes and Failure Cases

While GCA-Trans achieves robust performance in most scenarios, it still encounters challenges in highly ambiguous scenes. As illustrated in the qualitative analyses (Figure 4a,b, Figure 6 bottom row and Figure 9 bottom row), typical failure cases include the semantic misclassification of specific transparent categories and the misidentification of completely hollow regions or adjacent specular reflections as glass. Future work will explore incorporating stronger physical priors or cross-view multi-frame geometries to better resolve these complex ambiguities.

### 5.2. Customized Datasets for Specific Applications

Currently, our evaluations are primarily conducted on publicly available benchmark datasets. While these benchmarks are comprehensive, they may not fully encompass the specific challenges of targeted robotic applications, such as handling specialized experimental instruments in autonomous laboratories. Therefore, the lack of a customized dataset tailored to specific practical scenarios remains a limitation. In future work, we plan to collect and augment a large-scale, customized dataset featuring more diverse transparent object categories, complex physical interactions, and varying environmental conditions.

### 5.3. Model Efficiency

There remains room for improvement regarding computational efficiency, especially for deployment in resource-constrained, real-time robotic applications. Future work will focus on optimizing the network architecture to achieve a better balance between inference speed and segmentation performance.

### 5.4. Extreme Meteorological Conditions

The current evaluations are primarily conducted under standard imaging conditions. The robustness of our model under extreme weather (e.g., heavy rain, dense fog, or snow), which can severely degrade RGB signal quality, remains unexplored. We plan to extend our testing and adapt the model for such adverse scenarios.

### 5.5. Multimodal Integration

Our current approach relies exclusively on RGB imagery. To further enhance perception reliability, subsequent research will explore integrating multimodal inputs (e.g., polarization cameras or LiDAR) and incorporating uncertainty estimation. We aim to validate these comprehensive architectures on industrial datasets.

## 6. Conclusions

In this paper, we addressed the inherent imaging challenges of transparent object segmentation, specifically their lack of distinct texture and chromatic features, which causes their appearance to visually blend into the background. To overcome the architectural limitations of existing methods that often suppress weak surface details or struggle with significant scale variations, we proposed the Global Context-Aware Transformer (GCA-Trans). By designing a novel Multi-scale Context Mining (MCM) module, our approach effectively captures and integrates fine-grained local details, such as boundaries, subtle textures, and faint reflections, with coarse-grained global spatial context. This design facilitates consistent and robust feature representation for transparent objects across diverse scales. Extensive experiments demonstrate that GCA-Trans achieves competitive performance on four transparent object benchmark datasets. Quantitatively, our proposed method achieves significant improvements of 2.53% mIoU on Trans10K-v2, 2.1% IoU on RGB-D GSD, 2.2% IoU on GDD, and 1.9% IoU on GSD compared to previous leading methods. Furthermore, zero-shot evaluations on the VGSD-D dataset and general urban scenes (Cityscapes dataset) validate the promising effectiveness and potential for deployment of our model in complex and dynamic environments.

## Figures and Tables

**Table 1:** Summary of the evaluated datasets, detailing the data splits and the number of categories.

Dataset	Training Set	Validation Set	Testing Set	Categories
Trans10K-v2	5000	1000	4428	11
GDD	2980	-	936	1 (Binary)
GSD	3202	-	810	1 (Binary)
RGB-D GSD	2400	-	609	1 (Binary)

**Table 2:** Comparison of State-of-the-Art Models on Trans10K-v2 Sorted by mIoU. **Bold** formatting indicates the best performance. ↑ indicates that higher values are better, and ↓ indicates that lower values are better.

Method	GFLOPs ↓	Acc ↑	mIoU ↑	Category IoU ↑											
				Background	Shelf	Jar	Freezer	Window	Door	EyeGlass	Cup	Wall	Bowl	Bottle	Box
RGB-D GlassNet [3]	53.67	86.78	46.82	89.42	28.06	30.51	56.19	53.13	33.46	54.39	59.62	59.70	29.87	46.12	21.34
GDNet [6]	412.27	89.52	53.27	91.87	25.92	42.63	41.62	64.19	40.28	70.90	68.12	65.51	40.64	58.15	29.41
DUNet [39]	123.69	90.67	59.01	93.07	34.20	50.95	54.96	43.19	45.05	79.80	76.07	65.29	54.33	68.57	42.64
FCN [40]	42.23	91.65	62.75	93.62	38.84	56.05	58.76	46.91	50.74	82.56	78.71	68.78	57.87	73.66	46.54
DenseASPP [41]	36.20	90.86	63.01	91.39	42.41	60.93	64.75	48.97	51.40	65.72	75.64	67.93	67.03	70.26	49.64
OCNet [42]	43.31	92.03	66.31	93.12	41.47	63.54	60.05	54.10	51.01	79.57	81.95	69.40	68.44	78.41	54.65
PSPNet [35]	187.03	92.47	68.23	93.62	50.33	64.24	70.19	51.51	55.27	79.27	81.93	71.95	68.91	77.13	54.43
DANet [43]	198.00	92.70	68.81	93.69	47.69	66.05	70.18	53.01	56.15	77.73	82.89	72.24	72.18	77.87	56.06
DeepLabv3+ [44]	37.98	92.75	68.87	93.82	51.29	64.65	65.71	55.26	57.19	77.06	81.89	72.64	70.81	77.44	58.63
TransLab [4]	61.31	92.67	69.00	93.90	54.36	64.48	65.14	54.58	57.72	79.85	81.61	72.82	69.63	77.50	56.43
Trans2Seg [8]	49.03	94.14	72.15	95.35	53.43	67.82	64.20	59.64	60.56	88.52	86.67	75.99	73.98	82.43	57.17
Trans4Trans-S [9]	19.92	94.57	74.15	95.60	57.05	71.18	70.21	63.95	61.25	81.67	87.34	78.52	77.13	81.00	64.88
Trans4Trans-M [9]	34.38	95.01	75.14	96.08	55.81	71.46	69.25	65.16	63.96	83.84	88.21	80.29	76.33	83.09	68.09
TOSQ-128 [10]	41.48	95.34	76.63	96.07	47.56	78.94	66.63	**88.75**	75.18	69.13	90.69	72.96	87.18	81.47	64.98
TOSQ-256 [10]	43.15	95.53	77.47	96.22	52.08	**81.82**	66.65	88.70	**75.69**	69.14	91.03	68.38	**87.82**	81.76	67.32
TO-Former(B) [11]	117.739	95.603	77.35	96.79	49.94	76.93	76.77	65.88	66.38	87.81	90.52	81.63	82.15	84.81	68.53
**GCA-Trans-b3 (Ours)**	57.19	95.92	78.81	97.09	58.99	73.68	76.83	66.37	69.55	90.28	90.62	82.62	79.25	86.47	**73.96**
**GCA-Trans-b4 (Ours)**	73.48	**96.15**	**80.00**	**97.19**	**60.27**	76.78	**81.95**	69.30	70.61	**90.54**	**91.88**	**83.42**	82.42	**87.03**	68.66

**Table 3:** Quantitative results on the RGB-D GSD, GDD, and GSD datasets. **Bold** formatting indicates the best performance. ↑ indicates that higher values are better, and ↓ indicates that lower values are better.

Methods	Venue	RGB-D GSD				GDD				GSD			
		IoU↑	$F_{\beta }$↑	MAE↓	BER↓	IoU↑	$F_{\beta }$↑	MAE↓	BER↓	IoU↑	$F_{\beta }$↑	MAE↓	BER↓
GDNet [6]	*CVPR 2020*	0.468	0.631	0.119	19.25	0.814	0.909	0.097	8.83	0.790	0.869	0.069	7.72
EBLNet [5]	*ICCV 2021*	0.707	0.819	0.048	10.91	0.870	0.922	0.064	6.08	0.817	0.878	0.059	6.75
GSDNet [7]	*CVPR 2021*	0.714	0.822	0.048	9.73	0.881	0.932	0.059	5.71	0.836	0.903	0.055	6.12
RFENet [45]	*IJCAI 2023*	0.699	0.825	0.046	11.42	0.874	0.929	0.062	5.79	0.836	0.904	0.049	6.24
RGB-D GlassNet [3]	*AAAI 2025*	0.724	**0.853**	0.043	9.33	0.883	0.933	0.059	5.65	0.849	0.912	0.050	6.02
Trans4Trans-M [9]	-	0.697	0.789	0.053	11.98	0.888	0.931	0.057	5.453	0.818	0.880	0.059	7.26
PVT-v2 [13]	-	0.726	0.818	0.042	10.43	0.891	0.930	0.057	5.26	0.854	0.902	0.047	5.84
**GCA-Trans-b4**	-	**0.745**	0.8271	**0.037**	**9.09**	**0.905**	**0.939**	**0.047**	**4.43**	**0.868**	**0.912**	**0.041**	**4.73**

**Table 4:** Evaluation on VGSD-D test set [46]. **Bold** formatting indicates the best performance. ↑ indicates that higher values are better, and ↓ indicates that lower values are better.

Methods	IoU ↑	Accuracy ↑	BER ↓	MAE ↓
DeepLab [36]	0.705	0.845	16.67	0.155
Segformer [47]	0.744	0.855	13.50	0.145
SAM [48]	0.710	0.832	15.15	0.172
TVSD [49]	0.728	0.860	13.52	0.140
SC-Cor [50]	0.765	0.875	12.15	0.125
MirrorNet [51]	0.740	0.863	13.44	0.200
PMDNet [52]	0.765	0.879	11.47	0.181
VCNet [53]	0.751	0.873	12.17	0.168
VMD [54]	0.763	0.878	12.44	0.123
GDNet [6]	0.735	0.858	13.18	0.172
EBLNet [5]	0.764	0.868	13.25	0.134
GlassNet [55]	0.762	0.877	12.02	0.187
PGSNet [56]	0.703	0.846	15.11	0.156
VGSD-Net [46]	0.802	0.899	9.54	0.099
Ours (trained on GDD)	0.867	0.937	6.48	0.064
Ours (trained on GSD)	**0.896**	**0.952**	**4.93**	**0.050**
Ours (trained on RGB-D GSD)	0.867	0.939	6.12	0.063

**Table 5:** Quantitative comparison of per-category IoU on the Cityscapes validation set. **Bold** formatting indicates the best performance.

Category	Trans4Trans	TOSQ	Ours
road	**97.04**	96.04	97.03
sidewalk	71.78	76.67	**79.12**
building	86.44	87.79	**90.28**
wall	46.24	48.99	**52.15**
fence	31.30	44.31	**46.38**
pole	39.50	40.69	**60.32**
traffic light	42.49	22.40	**54.72**
traffic sign	53.62	60.14	**70.50**
vegetation	88.38	88.08	**91.62**
terrain	50.97	**60.53**	58.98
sky	92.61	89.57	**93.60**
person	66.14	67.01	**77.00**
rider	41.41	46.10	**53.72**
car	88.23	89.60	**92.76**
truck	50.30	**68.65**	54.37
bus	60.00	**74.19**	66.64
train	**57.18**	53.54	51.79
motorcycle	28.90	38.26	**47.05**
bicycle	58.95	55.66	**72.47**
mIoU	60.61	63.59	**68.97**

**Table 6:** Ablation study on the MCM module components and channel configurations. **Bold** and *italic* formatting indicates sub-headings within the table.

Variant	$C_{\mathrm{out}}$	Params (M)	GFLOPs	Acc (%)	mIoU (%)
***Component Analysis***					
ASPP	64	46.712	39.146	95.730	76.070
Only Dilated	64	49.375	57.137	95.775	77.937
Only Pooling	64	49.375	54.425	95.783	77.895
MCM	64	52.843	57.189	95.924	78.811
***Channel Analysis***					
GCA-Trans-b3	16	51.958	42.672	95.764	77.692
	32	52.253	47.511	95.819	78.751
	64	52.843	57.189	95.924	78.811
	128	54.025	76.546	95.749	78.743
	256	56.387	115.259	95.743	78.556
	512	61.112	192.686	95.609	77.636

**Table 7:** Inference efficiency analysis of GCA-Trans with different backbones. All metrics are measured with a batch size of 1 at 512×512 resolution on an NVIDIA 2080Ti GPU. *Italic* formatting indicates sub-headings. ↑ indicates that higher values are better, and ↓ indicates that lower values are better.

Backbone	Latency (ms) ↓	FPS ↑	GPU Memory (MB) ↓	RAM (MB) ↓
*PVT-v2 Backbones*				
b0	14.68	68.12	4239.66	3714.18
b1	16.39	61.00	4310.27	3721.47
b2	23.81	42.00	4381.77	3727.02
b3	32.82	30.47	4450.01	3736.21
b4	45.99	21.74	4496.33	3757.29
b5	57.75	17.32	4570.18	3765.49
*PVT-v1 Backbones*				
Tiny	15.97	62.63	4307.22	3716.99
Small	20.96	47.71	4374.85	3722.28
Medium	30.57	32.71	4426.47	3742.16
Large	41.80	23.92	4492.83	3747.05

## Data Availability

The datasets analyzed in this study (Trans10K-v2, RGB-D GSD, GDD, GSD, VGSD-D, and Cityscapes) are publicly available benchmark datasets. The code, pre-trained models, and experimental results presented in this study are openly available on GitHub at https://github.com/dongzujian/GCA-Trans (accessed on 2 May 2026).
